# Phylogenetic Profiling Analysis of the Phycobilisome Revealed a Novel State-Transition Regulator Gene in *Synechocystis* sp. PCC 6803

**DOI:** 10.1093/pcp/pcae083

**Published:** 2024-07-22

**Authors:** Tsukasa Fukunaga, Takako Ogawa, Wataru Iwasaki, Kintake Sonoike

**Affiliations:** Waseda Institute for Advanced Study, Waseda University, Tokyo 169-0051, Japan; Faculty of Education and Integrated Arts and Sciences, Waseda University, Tokyo 162-8480, Japan; Graduate School of Science and Engineering, Saitama University, Saitama 338-8570, Japan; Department of Integrated Biosciences, Graduate School of Frontier Sciences, The University of Tokyo, Chiba 277-0882, Japan; Faculty of Education and Integrated Arts and Sciences, Waseda University, Tokyo 162-8480, Japan

**Keywords:** Cyanobacteria (*Synechocystis* sp. PCC 6803), Phycobilisome, Phylogenetic profiling, Redox regulation, State transition, Universal stress protein

## Abstract

Phycobilisomes play a crucial role in the light-harvesting mechanisms of cyanobacteria, red algae and glaucophytes, but the molecular mechanism of their regulation is largely unknown. In the cyanobacterium, *Synechocystis* sp. PCC 6803, we identified *slr0244* as a phycobilisome-related gene using phylogenetic profiling analysis, a method used to predict gene function based on comparative genomics. To investigate the physiological function of the *slr0244* gene, we characterized *slr0244* mutants spectroscopically. Disruption of the *slr0244* gene impaired state transition, a process by which the distribution of light energy absorbed by the phycobilisomes between two photosystems is regulated in response to the changes in light conditions. The Slr0244 protein seems to act in the process of state transition, somewhere at or downstream of the sensing step of the redox state of the plastoquinone (PQ) pool. These findings, together with past reports describing the interaction of this gene product with thioredoxin and glutaredoxin, suggest that the *slr0244* gene is a novel state-transition regulator that integrates the redox signal of PQ pools with that of the photosystem I-reducing side. The protein has two universal stress protein (USP) motifs in tandem. The second motif has two conserved cysteine residues found in USPs of other cyanobacteria and land plants. These redox-type USPs with conserved cysteines may function as redox regulators in various photosynthetic organisms. Our study also shows the efficacy of phylogenetic profiling analysis in predicting the function of cyanobacterial genes that have not been annotated so far.

## Introduction

Photosynthesis is a light-energy conversion process that supports the global ecosystem in terms of energy. Because of its importance, photosynthesis has long been the subject of physiological, biochemical and genetic studies, resulting in the identification of the pigments and proteins involved in the reaction of photosynthesis. Most of the genes coding for proteins of the photosynthetic apparatus are now identified, at least in model organisms. Photosynthetic organisms, however, grow in ever-changing environments, and it is essential for them to regulate photosynthesis in response to environmental changes. For this purpose, there exist various regulatory mechanisms, e.g. the ones to optimize light harvesting, but the signaling and precise mechanisms for such regulation are still unclear.

The state transition is one such regulatory mechanism. Since photosynthetic electron transfer is driven by two photosystems, a balance between both photosystems is essential for efficient electron transfer. In state transitions, light energy absorbed by the light-harvesting antenna complex is allocated more to photosystem II (PSII) when photosystem I (PSI) is selectively excited (state 1) and to PSI when PSII is selectively excited (state 2) (Allen 1992). This allows the two photosystems to drive together for efficient electron transfer even when the light environment fluctuates. Light-harvesting chlorophyll proteins (LHCPs) serve as the antennae in land plants and green algae, while phycobilisomes serve in cyanobacteria, red algae and glaucophytes (Adir et al. 2020). Although the two antenna systems are totally different, i.e. LHCPs are intrinsic membrane proteins binding chlorophylls while phycobilisomes are soluble proteins binding open-ring tetrapyrroles, the regulatory mechanisms are quite similar; state 1 is induced by the oxidation of plastoquinone (PQ) pool and state 2 by the reduction of it. Since the PQ pool is located downstream of PSII and upstream of PSI in the photosynthetic electron transfer, it is reasonable that the redox signal of the PQ pool is used to regulate the allocation of light energy between the two photosystems. Several components are reported to be responsible for the regulation of state transition, but the signaling mechanism that bridges the redox state of the PQ pool and state transition is unknown.

In the case of cyanobacteria, several genes have been identified as the factors involved in the regulation of state transition. For example, the *rpa* (‘regulator of phycobilisome association’) genes have been reported to be involved in the regulation of state transition (Emlyn-Jones et al. 1999, Ashby and Mullineaux 1999b). The *psaK2* gene was also reported to be involved in the regulation of high-light acclimated cells of cyanobacteria (Fujimori et al. 2005). Since the *rpaC* gene is mainly expressed under low-light conditions while the *psaK2* gene is expressed under high-light conditions (Hihara et al. 2001), these two genes function under complementary conditions. In any event, the molecular function of the products of these genes have not been identified even now. The defects in the *apcD* and *apcE* genes are also reported to result in the absence of state transition (Ashby and Mullineaux 1999a), but the products of these genes are components of phycobilisome, so the function of these genes seems to be a structural one than the regulatory one. The difficulty in identifying the factors regulating state transition with their molecular mechanism can be partly ascribed to the ineffectiveness of conventional methods such as homology searches. Unlike the case of constituent proteins of phycobilisomes, alternative approaches have to be employed to identify the regulatory factors of state transition.

In the field of bioinformatics, various methods have been developed for the discovery of genes that are functionally related to specific genes. Phylogenetic profiling is one such method based on comparative genomics (Pellegrini et al. 1999, Kensche et al. 2008, Fukunaga et al. 2022). In this approach, an ortholog table is first constructed with genomes as rows, orthologs as columns and the presence or absence of orthologs as elements of the ortholog table. Subsequently, if two orthologs have similar distribution patterns across the genomes, they are assumed to be functionally related. Phylogenetic profiling analysis has been widely applied to eukaryotes as well as to prokaryotes, including cyanobacteria for elucidating the functions of genes whose function was unknown (Tabach et al. 2013, Kumagai et al. 2018). For example, Sato *et al*. identified genes associated with chloroplast endosymbiosis by the phylogenetic profiling analysis and experimentally examined growth defects in mutants of the identified genes (Sato et al. 2005). In another example, Beck *et al*. performed a phylogenetic profiling analysis on 77 cyanobacterial genomes, suggesting that several function-unknown genes may be involved in nitrogen fixation (Beck et al. 2018). Although the physiological function of these genes has not been sufficiently validated, these studies indicated the potential utility of the phylogenetic profiling analysis in cyanobacteria. Additionally, in *Synechocystis* sp. PCC 6803, the Fluorome database has been constructed, containing chlorophyll fluorescence data for about 750 mutants (Ozaki et al. 2007). This has the advantage that genes detected by the phylogenetic profiling analysis can be effectively examined without experiments to assess whether they can be involved in photosynthesis, one of the organism’s most important phenotypes. We therefore expected that the phylogenetic profiling analysis would be effective in finding novel genes relating to state transition.

In this study, we performed the phylogenetic profiling analysis for phycobilisomes and identified *slr0244* as a phycobilisome-related gene. Our physiological analysis showed that the mutants of the *slr0244* gene lacked the ability of normal state transition. Since the redox changes in the PQ pool can be induced by the addition of inhibitors of the electron transport chain without apparent state transition in the *slr0244* mutant, the *slr0244* gene seems to act somewhere at or downstream of the sensing step of the redox state of the PQ pool. These findings, together with past reports of the interaction of this gene product with thioredoxin or glutaredoxin (Li et al. 2007, Mata-Cabana et al. 2007), suggest that the *slr0244* gene is a novel state-transition regulator that integrates the redox signal of PQ pools with those on the PSI-reducing side. Our study also demonstrates the efficacy of phylogenetic profiling analysis in elucidating the function of cyanobacterial genes that have not been annotated so far.

## Results

### Phylogenetic profiling analysis to identify phycobilisome-related genes

In this study, we first constructed an ortholog table from publicly available cyanobacteria genomes with high genome assembly completeness. Through database searches and completeness checks, we identified 727 cyanobacterial genomes that met our criteria. However, constructing an ortholog table for this number of genomes would require a large amount of computational time. In addition, the phylogenetic profiling analysis is not expected to significantly improve the prediction performance when the number of genomes exceeds 100 (Škunca et al. 2015). Therefore, we opted to cluster the genomes using Mash (Ondov et al. 2016), an alignment-free tool for rapid estimation of genome distance, and selected 115 representative genomes. For the construction of the ortholog table, we employed SonicParanoid in the default mode (Cosentino and Iwasaki 2019), one of the most sensitive ortholog detection tools according to the Quest for Orthologs benchmarking service (Nevers et al. 2022).

We next performed the phylogenetic profiling analysis on the ortholog table to identify putative phycobilisome-related genes. We searched for genes that were absent in the eight genomes of three genera (*Prochlorococcus, Prochlorothrix* and *Acaryochloris*) that do not have the conventional phycobilisomes and are present in more than 80% of the remaining 107 genomes. Because the three genera are phylogenetically independent (Partensky et al. 2018), the genes thus listed (**Table 1**) should have been lost independently in the three genera. This phylogenetic independence increases the reliability of the phylogenetic profile analysis (Kensche et al. 2008). Five out of the top six genes in the list were components of the phycobilisome (*ssr3383, sll0928, slr2067, slr0335* and *slr1459*) and can be regarded as positive controls that supported the validity of the analysis. However, one remaining gene (*slr0244*) was functionally unknown. The Slr0244 protein is 284 amino acids in length and has two universal stress protein (USP) domains in tandem (The UniProt Consortium 2023). USPs are found in diverse taxa, including archaea, bacteria and plants. As its name suggests, USPs are assumed to mitigate environmental stress, but the details of their physiological functions have not been well understood (Chi et al. 2019). Analysis of the Fluorome database suggested that the *slr0244* gene is related to photosynthesis based on abnormal chlorophyll fluorescence kinetics of the *slr0244* mutant (**Supplementary Fig. S1**). Furthermore, co-expression analysis with the CyanoEXpress database suggested that the expression patterns of two components of the phycobilisome (*slr0335* and *slr2051*) were among the top 10 most similar to the expression pattern of *slr0244* (Hernández-Prieto et al. 2016). However, there is no known relationship between the *slr0244* gene and the phycobilisome. Therefore, we further investigated the function of the *slr0244* gene in this study by the physiological characterization of the disruption mutant of the *slr0244* gene. The mutant strain had been constructed by transposon mutagenesis (Ozaki et al. 2007). We confirmed that the mutant allele was completely segregated (**Supplementary Fig. S2A**).

**Table 1:** The ortholog list of phycobilisome-related genes detected by the phylogenetic profiling analysis

ORF names	Number of genomes	Gene function
*ssr3383*	106	Phycobilisome small core linker polypeptide
*sll0928*	106	Allophycocyanin-B
*slr2067*	106	Allophycocyanin alpha subunit
*slr0335*	105	Phycobilisome core-membrane linker polypeptide
*slr0244*	104	Hypothetical protein
*slr1459*	103	Phycobilisome core component
*sll1663*	100	Phycocyanin alpha phycocyanobilin lyase-related protein
*sll0021*	97	Probable exonuclease
*slr1784*	95	Biliverdin reductase
*slr1963*	92	Water-soluble carotenoid protein
*sll1004*	91	Hypothetical protein (dolichol-phosphate mannosyltransferase)
*slr1568*	91	Hypothetical protein
*slr2053*	91	Putative hydrolase
*sll0814*	90	Hypothetical protein
*sll0047*	86	Hypothetical protein YCF12 (PSII reaction center protein Ycf12)

The first, second and third columns represent the ORF names of *Synechocystis* sp. PCC 6803, the number of genomes possessing the ortholog and the gene function described in CyanoBase (Fujisawa et al. 2017), respectively.

### Measurements of the phycobilisome content

**Fig. 1:**
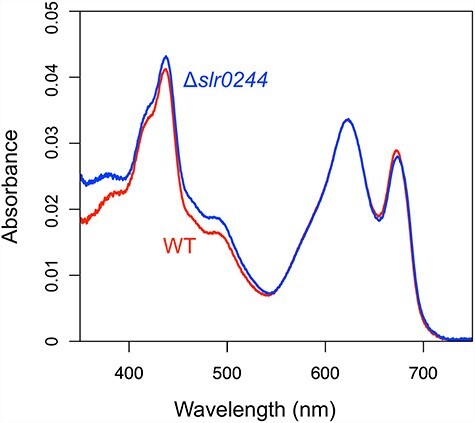
Absorption spectra of the cell suspension with OD_750_ = 0.2. The red and blue lines represent the WT and *slr0244* mutant, measured in triplicates, respectively.

We first examined the possibility that *slr0244* regulates the contents of phycobilisomes. The absorbance spectra of the intact cells of the wild type (WT) and the disruption mutant of *slr0244*, which was previously constructed through transposon mutagenesis, are shown in **Fig. 1**. At the same optical density (OD_750_ = 0.2), the absorbance at around 680 nm due to chlorophylls and that at around 620 nm due to phycobilisomes is similar between the WT and the *slr0244* mutant. Apparently, the phycobilisome contents as well as the chlorophyll contents were not much affected by the disruption of *slr0244*.

### Estimation of the state transition by low-temperature chlorophyll fluorescence spectra

**Fig. 2:**
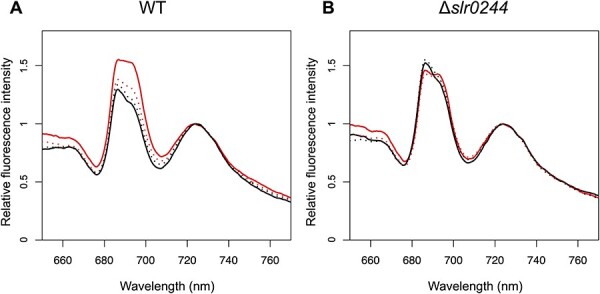
Chlorophyll fluorescence spectra at 77 K with phycocyanin excitation at 625 nm for (A) the WT and (B) the *slr0244* mutant. Before the measurements, the cyanobacterial cells were incubated in the light with DCMU (red solid line), in the light without DCMU (red dotted line), in the dark with KCN (black solid line) or in the dark without KCN (black dotted line). Each fluorescence spectrum was normalized at the peak of the PSI fluorescence. The averaged spectra from three biological replicates for each strain are shown.

We next examined the state transition, a regulatory mechanism by which light energy absorbed by the phycobilisome is distributed between the two photosystems in response to the changes in light conditions. State transition is known to be regulated by the redox condition of the PQ pool located between PSI and PSII. When the PQ pool is oxidized by preferential excitation of PSI (so-called ‘state 1’), light energy absorbed by antenna pigments is allocated to PSII. For example, when the WT cells were illuminated in the presence of 3-(3,4-dichlorophenyl)-1,1-dimethylurea (DCMU), an inhibitor of PSII, the PQ pool was oxidized leading to the higher allocation of light energy to PSII, which was reflected in the high PSII fluorescence at 685–695 nm determined at low temperature (**Fig. 2A**). On the other hand, when the PQ pool is reduced by the preferential excitation of PSII (so-called ‘state 2’), light energy is allocated to PSI. In the case of cyanobacteria, the PQ pool of the WT cells can be also reduced by incubation in the dark in the presence of potassium cyanide (KCN), an inhibitor of respiratory terminal oxidase, since the PQ pool is shared between photosynthetic and respiratory electron transport chains (Aoki and Katoh 1982, Peschek and Schmetterer 1982). The condition, therefore, induces the transition to state 2 in the WT cells, which is reflected in the low PSII fluorescence in the low-temperature chlorophyll fluorescence spectrum (**Fig. 2A**). Through this change in PSII fluorescence, we analyzed the state transition of the *slr0244* mutant. Only minor differences were observed in the chlorophyll fluorescence spectra of the *slr0244* mutant between state 1 and state 2 inducing conditions (**Fig. 2B**), suggesting the lack of state transition in the mutant.

To exclude the possibility that the observed phenotype was derived from secondary mutations in the mutant, we constructed another mutant of the *slr0244* gene and analyzed its state transition. We amplified the *slr0244* gene region in the first mutant strain by PCR and transformed the WT cells with the PCR products. After confirming that the mutant allele was also completely segregated in this new mutant strain (**Supplementary Fig. S2B**), we determined the chlorophyll fluorescence spectra of this mutant (**Supplementary Fig. S3**). The mutant showed only a minor change in the PSII fluorescence, confirming that the phenotype is certainly brought about by the disruption of the *slr0244* gene.

### Estimation of the state transition by pulse amplitude modulation chlorophyll fluorometry at room temperature

Although the lack of state transition in the *slr0244* mutants can be explained by the defect in the regulatory mechanism of state transition, the phenotype can be also brought about by the defect in the regulatory mechanism of the redox state of the PQ pool. We next investigated which of the two possibilities was the case. The chlorophyll fluorescence yield (F level) of cyanobacterial cells determined by pulse amplitude modulation (PAM) chlorophyll fluorometry mainly reflects the redox state of the PQ pool with a smaller contribution of state transition (Schreiber et al. 1995, Ogawa and Sonoike 2016). When a saturating pulse is applied, the chlorophyll fluorescence yield increases due to the full reduction of *Q*_A_, which is in equilibrium with the PQ pool, and this level (Fm′ level) reflects solely state transition. Through the changes in F level and Fm′ level, we can analyze the relationship between state transition and PQ redox condition.

**Fig. 3:**
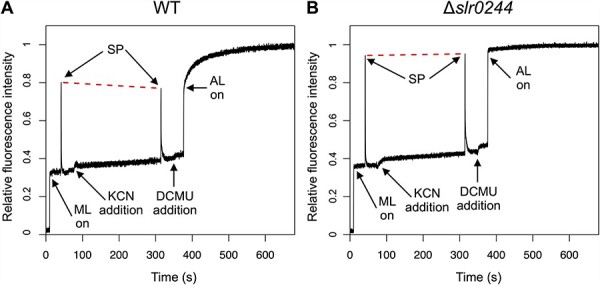
The kinetics of chlorophyll fluorescence determined by PAM chlorophyll fluorometer for (A) the WT and (B) the *slr0244* mutant. A typical trace for each strain is shown from the experiments of three biological replicates. ML, AL and SP stand for the measuring light, the actinic light and the saturating pulse, respectively. The red dashed lines are drawn to connect the two peaks of the fluorescence upon saturating pulse irradiation for comparison. Each fluorescence spectrum was normalized at the level of Fm.

Upon addition of 0.2 mM KCN to the cell suspensions, F level gradually increased due to the reduction of *Q*_A_ and the PQ pool in the WT cells as well as in the *slr0244* mutant cells (**Fig. 3**). Subsequent application of the saturating pulse induced the increase of fluorescence level to Fm′, but the level was lower than that before the addition of KCN in the case of the WT cells (**Fig. 3A**), indicating the transition to state 2. On the other hand, the Fm′ level did not change before and after the addition of KCN in the case of the *slr0244* mutant cells (**Fig. 3B**). Thus, the state transition is impaired in the mutant, even though the redox change of the PQ pool is normal.

The lack of state transition was also confirmed by the fluorescence changes induced under the DCMU/light condition. Upon the start of actinic illumination (53 μmol m^−2^ s^−1^) in the presence of 10 μM DCMU, the F level sharply increased to the level near the Fm′ level before the addition of DCMU both in the WT cells and in the *slr0244* mutant cells, reflecting the reduction of *Q*_A_ and the PQ pool. In the case of the WT, the F level subsequently increased to the Fm level, which was much higher than the Fm′ level, reflecting the slow transition to state 1. In the case of the mutant, however, this subsequent increase in fluorescence was absent, and the fluorescence levels were similar between Fm′ and Fm, indicating that the *slr0244* mutant cells were always in state 1 irrespective of the redox state of the PQ pool.

### Analysis of the redox state of the NADPH

The above results indicate that the redox changes in the PQ pool cannot induce state transition in the *slr0244* mutant. Apparently, the sensing mechanism of the redox state of the PQ pool or downstream signal transduction or actual mechanism of state transition should be impaired. On the other hand, we cannot deny the possibility that some signal other than the redox state of the PQ pool overrides it. As a point of redox regulation in photosynthesis, the reducing side of PSI is well known. The redox states of electron acceptors for PSI, ferredoxin and nicotinamide adenine dinucleotide phosphate (NADP^+^/NADPH for oxidized/reduced form), regulate the activities of many enzymes through the thioredoxin system in cyanobacteria as well as in land plants (Lindahl and Kieselbach 2009). Therefore, we analyzed the redox condition of the reducing side of PSI by monitoring the NADPH fluorescence.

**Fig. 4:**
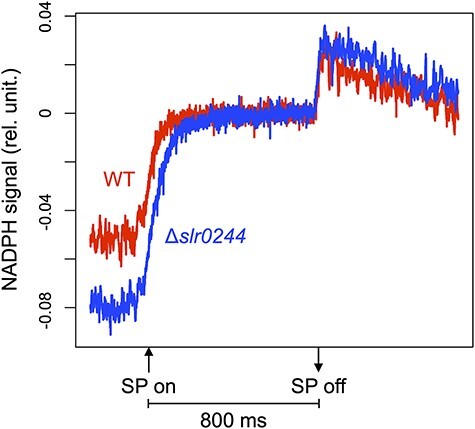
The dark-to-light induction kinetics of the NADPH fluorescence under the saturating pulse (SP) for 800 ms. The red and blue lines represent the WT and *slr0244* mutant, respectively. The average values from three biological replicates for each strain are shown.

NADPH absorbs UV-A light and emits blue fluorescence, while its oxidized form, NADP^+^, is not fluorescent. When a saturating pulse was applied to dark acclimated cyanobacterial cells, the NADPH fluorescence increased due to the photoreduction of NADP^+^ (**Fig. 4**). This dark-to-light induction kinetics of the NADPH fluorescence in the WT and the *slr0244* mutant are more or less similar, suggesting the typical photoreduction of NADP^+^ by photosynthetic electron transport in the WT cells as well as in the *slr0244* mutant cells. Compared to the WT cells, the fluorescence increase in the *slr0244* mutant cells was larger, indicating that NADPH was more oxidized in the mutant in the dark.

**Fig. 5:**
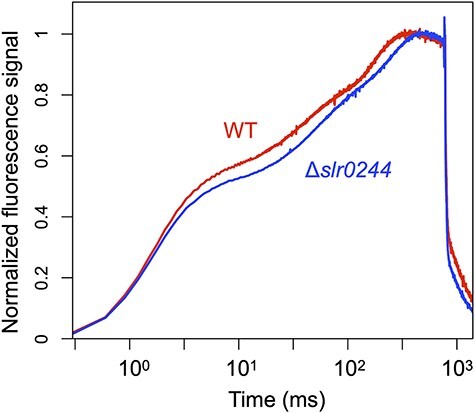
The dark-to-light induction kinetics of the chlorophyll fluorescence under the saturating pulse for 800 ms. The red and blue lines represent the WT and *slr0244* mutant, respectively. The fluorescence level at each time point (Ft) was normalized using the formula (Ft−Fo)/(Fp−Fo). Here, Fo represents the average fluorescence level before the saturating pulse and Fp denotes the peak fluorescence level under the saturating pulse. The time is shown in logarithmic scale. The average values from three biological replicates for each strain are shown.

The redox state of NADPH was further assessed by measuring chlorophyll fluorescence under the same conditions used for the NADPH fluorescence measurement. During the transition from dark to light, the chlorophyll fluorescence shows a characteristic induction curve called the OJIP curve. In the case of cyanobacterial cells, the increase in the fluorescence during the first 70 ms of saturating pulse illumination mainly reflects the redox state of the PQ pool in the dark. On the other hand, the redox state of NADPH in the dark was mainly reflected in the 70–800 ms region of the saturating pulse (Ogawa et al. 2021). Compared with the WT cells, the fluorescence induction kinetics of the *slr0244* mutant shows lower fluorescence (i.e. slower rise) in both the 0–70 ms region and 70–800 ms region (**Fig. 5**). The results indicate that, in the dark-acclimated mutant cells, the redox states of both PQ pool and NADPH are in more oxidized condition compared to the WT cells.

### Expression analysis of the downstream genes of *slr0244*

We finally examined the possibility that the phenotypes in the *slr0244* mutant were due to changes in the expression levels of the downstream genes of *slr0244*, rather than the deletion of the *slr0244* gene itself. We investigated the expression levels of two downstream genes, *psbM* and *slr0245*, using semi-quantitative reverse transcription (RT)-PCR, and compared their expression levels between the WT and the mutant. As a result, there were no significant differences in the expression levels of these two genes (**Supplementary Fig. S4**). In addition, based on the number of PCR cycles required for quantification, the expression level of *slr0245* was estimated to be very low in both strains. Previous research by Kopf *et al*. showed that *slr0244* was a single transcription unit and the expression level of *slr0245* was very low (Kopf et al. 2014), consistent with our findings. These results suggest that the phenotypes in the *slr0244* mutant were caused by the deletion of *slr0244* itself, rather than the positional effect of *slr0244* deletion.

## Discussion

### Phylogenetic strategy to seek the regulators of state transition

In this study, we searched for genes that are related to phycobilisome function using phylogenetic profiling analysis. One of the candidates obtained through this analysis, the *slr0244* gene with no known function, was shown to play a role in regulating energy distribution between PSI and PSII by state transition (**Figs. 1**–**3**). This is the first study in cyanobacteria in which the gene function predicted by the phylogenetic profiling analysis is validated physiologically.

In our phylogenetic profiling analysis, the *slr0244* ortholog was present in 104 out of 107 genomes of organisms having conventional phycobilisomes. The three genomes lacking the *slr0244* ortholog were genomes of *Halomicronema hongdechloris* C2206, *Snowella* sp. and *Cyanothece* sp. SIO1E1. The latter two genomes are metagenome-assembled ones (MAGs) and the absence of the *slr0244* ortholog could be ascribed to errors in the construction of MAGs. On the other hand, the genome of *H. hongdechloris* has been individually sequenced (Chen et al. 2019) and is likely to indeed lack the *slr0244* ortholog. *H. hongdechloris* has the unique feature of having chlorophyll *f*, which absorbs far-red light, in addition to phycobilisomes and chlorophyll *a* (Chen et al. 2012). Interestingly, *H. hongdechloris* undergoes a unique state transition that distributes light energy between PSII containing chlorophyll *f* and the other PSII without chlorophyll *f* (Schmitt et al. 2020). This unique state transition system may be related to the absence of the *slr0244* ortholog in *H. hongdechloris*. It must be noted, however, that some species like *Chlorogloeopsis fritschii* PCC 6912 have chlorophyll *f* (Airs et al. 2014) as well as the *slr0244* ortholog in their genomes.

Among the genes without any known function, the *slr0244* gene was ranked highest in the list of candidate orthologs obtained by the phylogenetic analysis (**Table 1**). The other genes with the annotation, ‘hypothetical protein’, such as the *slr1568* and *sll0814* genes, and those with enzymatic annotations, ‘probable exonuclease’ or ‘putative hydrolase’, such as the *sll0021* and *slr2053* genes, are also candidates for the regulatory factors of phycobilisome function. In this context, it is worth mentioning that the *slr1963* gene codes orange carotenoid protein, which is a factor regulating the energy dissipation in the phycobilisome (Gwizdala et al. 2011). It must be noted, however, that the lower the rank of genes in the list, the higher the risk of detecting genes unrelated to the phycobilisomes.

While we focused on phycobilisomes for our phylogenetic profiling analysis, we could also target genes associated with other cyanobacterial functions. However, due to the characteristics of the phylogenetic profiling analysis, we are unable to investigate the functions that are universally present in almost all cyanobacteria, such as core components of photosynthesis, with this method but can only focus on specific functions whose presence or absence varies among cyanobacterial species. For example, analyzing characteristics such as whether a species is unicellular or multicellular and whether a species has nitrogen-fixing capacity would be strong targets (Chen et al. 2022). In addition, other bioinformatics-based methods for predicting gene function, such as co-expression analysis (Obayashi et al. 2022) and protein interactome analysis (Bryant et al. 2022), are expected to empower the phylogenetic profiling in elucidating the function of function-unknown genes.

### Mechanism of cyanobacterial state transition

Since state transition is induced by the redox changes in the PQ pool, the mutants with different PQ redox often show apparent defects in state transition. For example, the mutant of the *ndhF1* gene, which encodes a subunit of the respiratory NAD(P)H dehydrogenase (NDH-1) complex, shows the phenotype of the oxidized PQ pool due to the lack of electron flow from NDH-1 to the PQ pool and is thus locked in state 1 in the dark, leading to the apparent lack of ability of state transition (Ogawa et al. 2013). A similar phenotype was observed in the mutant of the *gnd* gene, which encodes the enzyme of the oxidative pentose phosphate pathway that produced NADPH in cyanobacteria (Ogawa et al. 2021). In these cases, however, state transition can be induced by the addition of KCN, an inhibitor of terminal oxidase of respiratory electron transport that oxidized the PQ pool in the dark. This is contrary to the case of the *slr0244* mutant, in which state transition could not be induced even in the presence of KCN (**Figs. 2** and **3**). Although the redox state of the PQ pool or the NADPH pool in the *slr0244* mutant was somewhat oxidized in the dark compared with the case of WT (**Figs. 4** and **5**), this is not the cause of the absence of state transition in the *slr0244* mutant, suggesting that the mutant is insensitive to the redox changes in the PQ pool.

The *rpa* mutant has been reported to be incapable of state transition, but it was not shown whether the phenotype is due to the lack of regulation of state transition or of the redox regulation of the PQ pool (Emlyn-Jones et al. 1999, Ashby and Mullineaux 1999b). According to the Fluorome database, the database of the induction kinetics of chlorophyll fluorescence for about 750 mutants of *Synechocystis* sp. PCC 6803 (Ozaki et al. 2007, Ozaki and Sonoike 2009), the chlorophyll fluorescence kinetics of the *rpaB* mutant is similar to that of the *slr0244* mutant, suggesting a potential relationship between these two genes. On the other hand, AlphaFold-multimer in ColabFold (Jumper et al. 2021, Mirdita et al. 2022) showed low scores on predicted local distance difference test (pLDDT) at the predicted interaction site between *rpaB* and *slr0244*, suggesting that they may not physically interact. The similarity in chlorophyll fluorescence kinetics between the two mutants may be ascribed to the more oxidized PQ pool in the mutants, but further studies are required to elucidate the actual relationship between the *slr0244* and *rpaB* gene products. In the case of some phycobilisome-related genes (the *apcD* and *apcE* genes) and a PSI-related gene (the *psaK2* gene), which were also reported to be involved in state transition (Ashby and Mullineaux 1999a, Fujimori et al. 2005), the effects of the mutations may be a more direct one.

### Characteristics of the Slr0244 protein

**Fig. 6:**
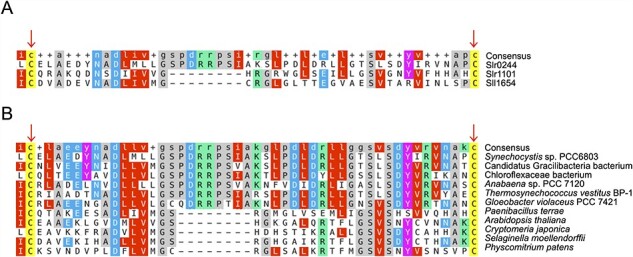
Multiple alignment results of the USP regions with two conserved cysteines in (A) the USPs in *Synechocystis* sp. PCC 6803 and (B) the USPs in the other cyanobacteria, bacteria and land plants.

Although we used phylogenetic and physiological approaches to reveal the physiological mechanism of state transition in this study, some close examinations of the sequence of the *slr0244* gene and its homolog have given some information about the biochemical role of the Slr0244 protein. The Slr0244 protein has two USP domains in tandem, and there are nine other genes with one or two in tandem interactome analysndem USP in the genome of *Synechocystis* sp. PCC 6803. The analysis with a multiple alignment program (MAFFT) (Katoh *et al*. 2019) showed that the second USP domain of the Slr0244 protein has two cysteine residues that are conserved in the USP domains of the Slr1101 and Sll1654 proteins (**Fig. 6A**). Furthermore, these two cysteines are also conserved in some of the USP domains of land plants, including angiosperms (e.g. *Arabidopsis thaliana*), gymnosperms (e.g. *Cryptomeria japonica*), pteridophytes (e.g. *Selaginella moellendorffii*) and bryophytes (e.g. *Physcomitrella patens*), as well as other cyanobacteria and bacteria (**Fig. 6B**). Except for *Paenibacillus* and *Gracilibacteria*, these organisms are photosynthetic. Note that we could not find the USP domain with these two conserved cysteine residues in archaea, *Escherichia coli*, and *Bacillus subtilis*. Since land plants do not have phycobilisomes, those proteins found in land plants cannot be regarded as orthologs of the Slr0244 protein and should have different roles from that of the Slr0244 protein. The ortholog analysis using the SonicParanoid in the default mode also suggested that there were no orthologs of the Slr0244 protein in these land plants. Nevertheless, we assume that this domain with two cysteine residues should have a role in redox regulation both in cyanobacteria and in land plants. Concerning this point, the Slr0244 protein was listed as one of the targets of thioredoxin (Mata-Cabana et al. 2007) and glutaredoxin (Li et al. 2007). At least in the case of cyanobacteria, it may be assumed that the proteins with a USP domain containing two conserved cysteines serve for the regulation through the redox condition of the PSI acceptor side. This suggests that the USP, which was involved in stress responses in other microbes, has evolutionarily acquired novel functions in the mechanisms of photosynthesis within cyanobacteria.

Because the state transition is regulated by the redox state of the PQ pool, it was unexpected that the regulatory factor of the state transition may be regulated by the redox state of the PSI acceptor side as well. In this regard, it may be necessary to consider that the state transition of cyanobacteria is induced not only by the changes in light quality (PSI excitation light/PSII excitation light) but also by the changes in light quantity (low light/high light). Furthermore, the *rpaB* gene, which was initially identified as a *r*egulator of *p*hycobilisome *a*ssociation, was shown in high-/low-light acclimation as a response regulator of the two-component system in signal transduction (Muramatsu and Hihara 2012). It is tempting to assume that the light quality signal as the redox state of the PQ pool and the light quantity signal as the redox state of the PSI acceptor side are integrated through the action of the Slr0244 protein.

Interestingly, the *Arabidopsis* proteins with the USP domain containing two conserved cysteines are annotated as ‘adenine nucleotide alpha hydrolases-like superfamily proteins’, and some of the proteins are shown to bind adenine nucleotide (Kim *et al*. 2015). In addition, using AlphaFold3 (Abramson et al. 2024) to find the binding ligands for the predicted structure of the Slr0244 protein, it seems that the Slr0244 protein also binds ATP (**Supplementary Fig. S5**). The ATP-binding region is in the first USP domain, while the two conserved cysteines are in the second USP domain. Thus, the functions of the two domains may differ. The function of this ATP is unknown in cyanobacteria as well as in *Arabidopsis*, but we assume some role as a molecular switch in these proteins. At any rate, the mechanism of the regulation of state transition seems to be very complex, and extensive biochemical investigation will be necessary for the elucidation of the entire mechanism.

## Materials and Methods

### Construction of the ortholog table

We first downloaded all 926 annotated cyanobacterial genomes and entire protein products with the assembly level of ‘complete’, ‘chromosome’ or ‘scaffold’ from the NCBI assembly database (Kitts *et al*. 2016) on 1 July 2022, except for the annotation of the *Synechocystis* sp. PCC 6803 genome, which was adopted from CyanoBase (Fujisawa et al. 2017) to ensure consistency with other databases used in this study. To exclude incomplete or symbiotic cyanobacterial genomes from the analysis, we applied the genome assembly completeness checking tool (BUSCO with the ‘cyanobacteria_odb10ʹ model) (Manni et al. 2021) to the downloaded genomes and retained 727 genomes with a completeness score of 90% or higher (**Supplementary Data 1**). We then performed clustering of the genomes to select representative genomes for constructing the ortholog table. The distances between the genomes were calculated exhaustively using Mash (Ondov et al. 2016), and hierarchical clustering was performed using the single linkage method. The clusters were divided at a branch length of 0.2. This procedure resulted in 115 clusters and one representative genome was selected from each cluster (**Supplementary Data 2**). We selected the representative genomes based on the oldest registration date to the NCBI assembly database within each cluster. Finally, we constructed the ortholog table by applying SonicParanoid in the default mode (Cosentino and Iwasaki 2019) to the entire protein products of the selected 115 genomes.

### Phylogenetic profiling analysis

While most cyanobacteria have the conventional phycobilisomes, three genera of non-symbiotic cyanobacteria lack the conventional phycobilisomes: *Prochlorococcus* (Chisholm et al. 1992), *Prochlorothrix* (Burger-Wiersma et al. 1986) and *Acaryochloris* (Miyashita et al. 1997). The 115 genomes used in our analysis included eight genomes from these three genera: *Prochlorococcus marinus* subsp. *marinus* str. CCMP1375, *Prochlorococcus marinus* subsp. pastoris str. CCMP1986, *Prochlorococcus marinus* str. MIT9313, *Prochlorococcus marinus* str. NATL2A, *Prochlorococcus marinus* str. MIT9211, *Prochlorothrix hollandica* PCC9006 = CALU1027, *A. marina* MBIC11017 and *A. thomasi* RCC1774. In this study, we selected genes that were absent in all eight genomes of the three genera but were present in more than 80% of the remaining 107 genomes, as putative phycobilisome-related genes.

### Culture conditions and strains

The WT and the *slr0244* mutants of *Synechocystis* sp. PCC 6803 cells were cultured at 30℃ in BG-11 medium (Allen 1968) buffered with 20 mM 2-[[1,3-Dihydroxy-2-(hydroxymethyl)propan-2-yl]amino]ethanesulfonic acid (TES) and KOH (pH 8.0). The cells were bubbled with air under continuous growth light at 50–80 µmol m^−2^ s^−1^ for 24 h. We added chloramphenicol at 25 µg ml^−1^ to the culture medium for the mutants. We used two *slr0244* mutant strains. The first mutant strain had been constructed by transposon mutagenesis, and a chloramphenicol-resistance cassette was inserted into the *slr0244* gene (Ozaki et al. 2007). Unless otherwise stated, we used this strain as the default mutant strain. To exclude the possibility that the observed phenotypes in the mutant were due to secondary mutations, we constructed the second mutant strain. We amplified the *slr0244* gene region including the chloramphenicol-resistance cassette in the first mutant strain by PCR. The forward primer was 5ʹ-TATACGCAGATTCTGGCACC-3ʹ and the reverse primer was 5ʹ-TTAGATCCCTTCTTTGCGAG-3ʹ. We then transformed the WT cells with the PCR products, and the segregation of the mutant allele was checked by PCR genotyping of the mutant strains.

### Absorbance spectra

We determined the absorbance spectra of the intact cells using a spectrophotometer (V-650, JASCO, Tokyo, Japan) with an integrating sphere unit (ISV-722, JASCO). The optical density of the cell suspension was determined at 750 nm (OD_750_) without the integrating sphere and adjusted to 0.2 before the measurements of the absorbance spectra. We presented the average spectra of three biological replicates.

### Chlorophyll contents

Chlorophylls in cells were extracted by methanol, and the absorbance of the methanol solution was determined by a spectrophotometer (V-650, JASCO). The chlorophyll concentration was calculated as described in Grimme and Boardman (1972).

### Evaluation of state transition by low-temperature chlorophyll fluorescence spectra

We estimated the ability of state transition of the cyanobacterial cells by chlorophyll fluorescence spectra determined at 77 K using a fluorescence spectrometer (FP-8500, JASCO) with a low-temperature attachment (PU-830, JASCO) as reported earlier (Ogawa et al. 2013). Before the measurements, the cells were incubated for 15 min at room temperature in one of the following conditions: (i) dark condition, (ii) the dark with 0.1 mM KCN, (iii) light (98 µmol m^−2^ s^−1^) condition and (4) the light with 10 µM DCMU. The chlorophyll concentration of the cell suspension was adjusted to 2 µg ml^−1^. The fluorescence spectra were normalized at the peak of PSI fluorescence, and the averaged spectra of three biological replicates were presented.

### Evaluation of state transition by chlorophyll fluorescence at room temperature

We also estimated the ability of state transition of the cyanobacterial cells by PAM chlorophyll fluorescence measurements at room temperature using a fluorometer (Multi-Color-PAM, Walz, Effeltrich, Germany). We used blue (440 nm) measuring light to determine the chlorophyll fluorescence and red (625 nm) actinic light for the excitation of phycobilisomes. Cell suspensions were acclimated in the dark for 15 min before the measurements. The measuring procedure is as follows: turning on of measuring light—30 s—a saturating pulse—30 s—addition of 0.2 mM KCN to the samples—120 s—a saturating pulse—30 s—addition of 10 µM DCMU—30 s—actinic light illumination for 5 min. Fm′ is the fluorescence level upon the saturating pulse under each condition. Fm is the maximum fluorescence level upon saturating pulse in the presence of DCMU under actinic light. A magnetic stirrer was placed in the cuvette to ensure the mixing of the cell suspensions. The chlorophyll concentration of the cell suspensions was adjusted to 2 µg ml^−1^.

### Simultaneous measurements of NADPH and chlorophyll fluorescence

We simultaneously measured NADPH and chlorophyll fluorescence using a fluorometer (Dual-PAM-100, Walz) as described in Ogawa et al. (2021). The chlorophyll concentration in the cell suspension was 10 µg ml^−1^. Before the measurements, samples were exposed to 16 μmol m^−2^ s^−1^ red (620 nm) light for 1 min, followed by dark acclimation for 15 min. The kinetics determined for the three biological replicates were averaged and presented.

### Semi-quantitative RT-PCR analysis

We performed reverse transcription on the total RNA extracted from the WT and mutant cells using TRIzol reagent (Thermo Fisher Scientific, Waltham, MA, USA). For cDNA synthesis, 1 μg of the total RNA was reverse transcribed using a ReverTra Ace qPCR RT kit (TOYOBO, Tokyo, Japan) according to the manufacturer’s instructions. Semi-quantitative RT-PCR was performed using the resulting cDNA as a template. The PCR targets were *psbM* and *slr0245*, downstream genes of *slr0244*, and *rnpB*, a control gene (Wang et al. 2004). Primers for each gene are listed in **Supplementary Table S1**. The PCR cycle numbers for *psbM, slr0245* and *rnpB* were 26, 31 and 23, respectively. The expression levels of each gene were quantified by lane profile analysis with ImageJ and normalized to the expression level of *rnpB* for comparisons between the strains.

## Supplementary Material

pcae083_Supp

## Data Availability

The data in the study are available in the article and the **Supplementary materials**.
