# Supplementary material for: Phylogenetic Profiling Analysis of the Phycobilisome Revealed a Novel State-Transition Regulator Gene in Synechocystis sp. PCC 6803
Source: Plant Cell Physiol. 2024 Jul 22;65(9):1450–60. doi: 10.1093/pcp/pcae083 (PMC11447641; doi:10.1093/pcp/pcae083)
Supplement: pcae083_Supp [file pcae083_supp.zip › suppl_data/pcp-2024-e-00052-File009.docx]

**Plant and Cell Physiology Supplementary Materials**

Article title: Phylogenetic Profiling Analysis of the Phycobilisome Revealed a Gene Encoding Novel State-Transition Regulator in *Synechocystis* sp. PCC 6803

Authors: Tsukasa Fukunaga, Takako Ogawa, Wataru Iwasaki and Kintake Sonoike*


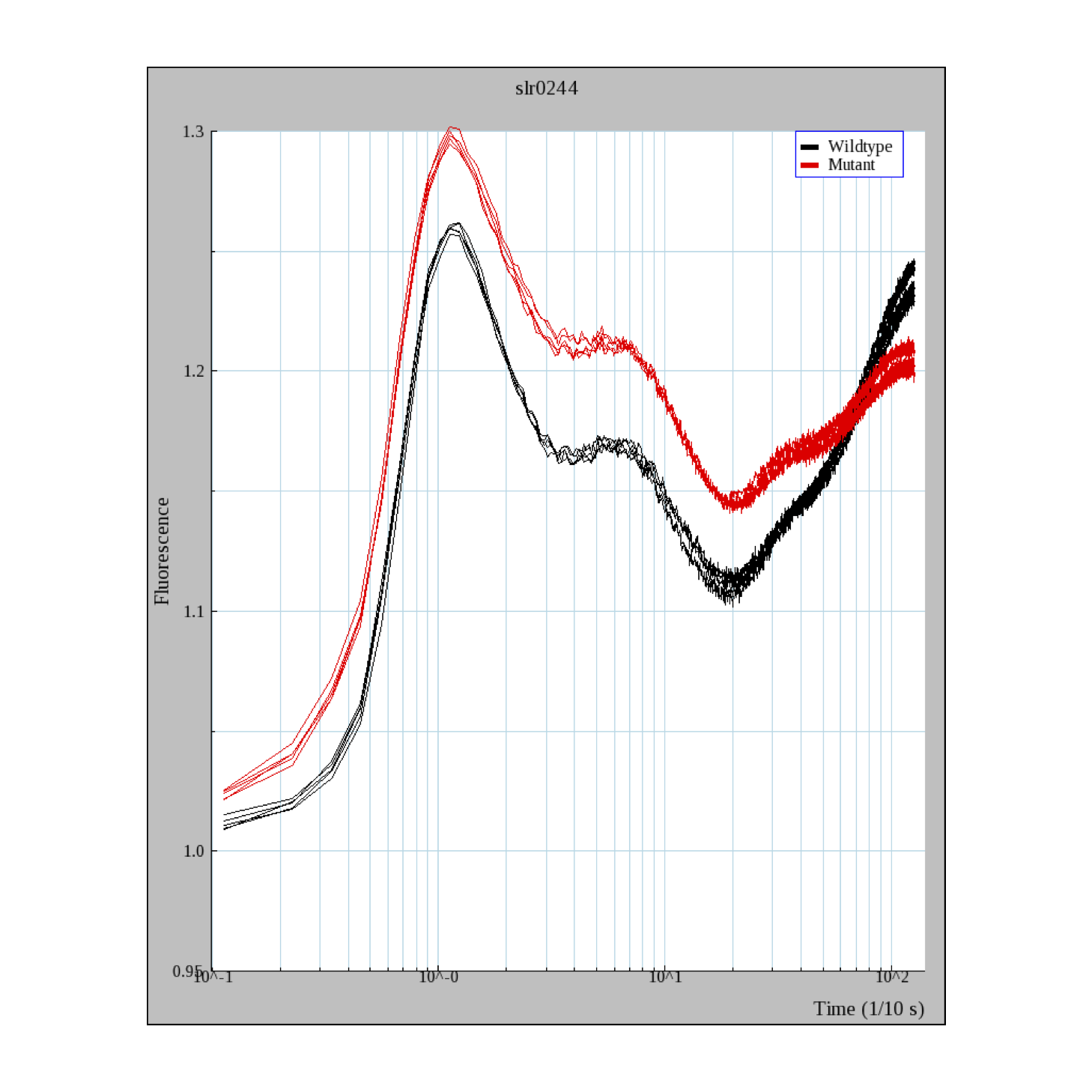


**Fig. S1** The kinetics of the chlorophyll fluorescence of the WT (black lines) and the *slr0244* mutant (red lines) cells grown under low light conditions taken from the Fluorome database. The time is shown in logarithmic scale (X-axis) and the fluorescence signal is normalized at the initial value (Y-axis).

**
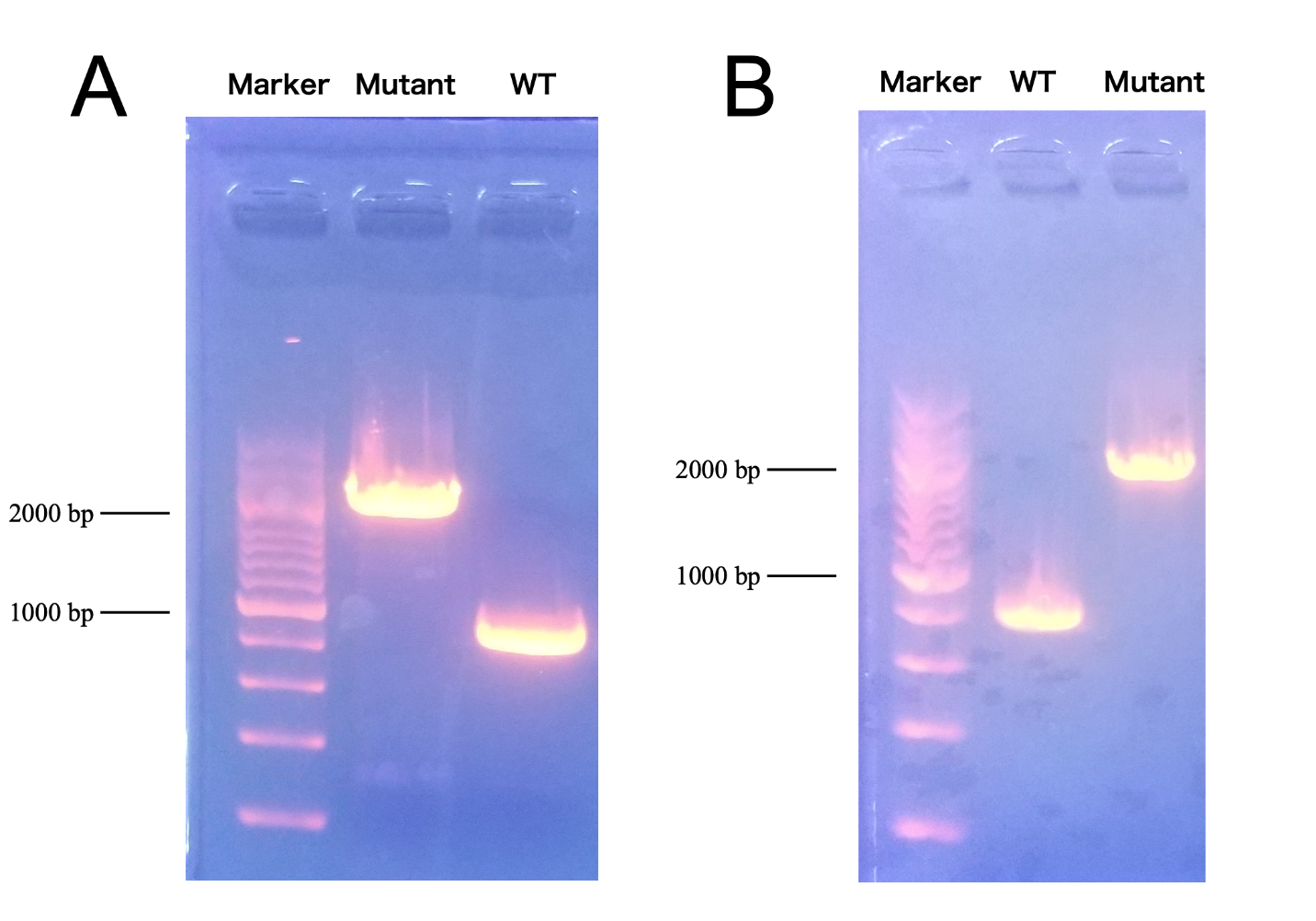
**

**Fig. S2** PCR genotyping of (A) the first mutant strain and (B) the newly constructed *slr0244* mutant strain. The *slr0244* region was amplified by PCR using genomic DNA from the WT or the newly constructed mutant strain as templates. The mutant allele includes a chloramphenicol-resistance cassette region.


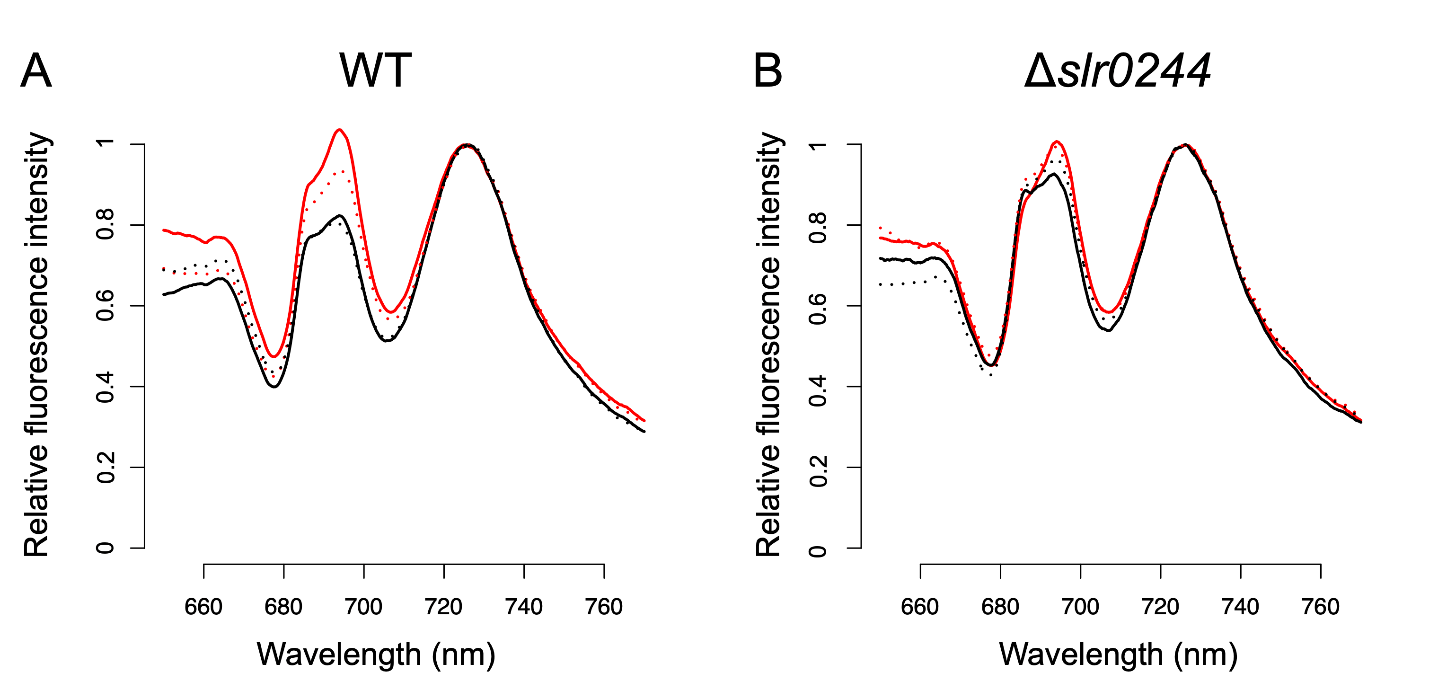


**Fig. S3** 77K chlorophyll fluorescence spectra with phycocyanin excitation at 625 nm for (A) the WT and (B) the newly constructed *slr0244* mutant. Before the measurements, cyanobacterial cells were incubated in the light with DCMU (red solid line), in the light without DCMU (red dotted line), in the dark with KCN (black solid line), or in the dark without KCN (black dotted line). Each fluorescence spectrum was normalized at the peak of the PSI fluorescence. The averaged spectra from three biological replicates for each strain are shown.

**
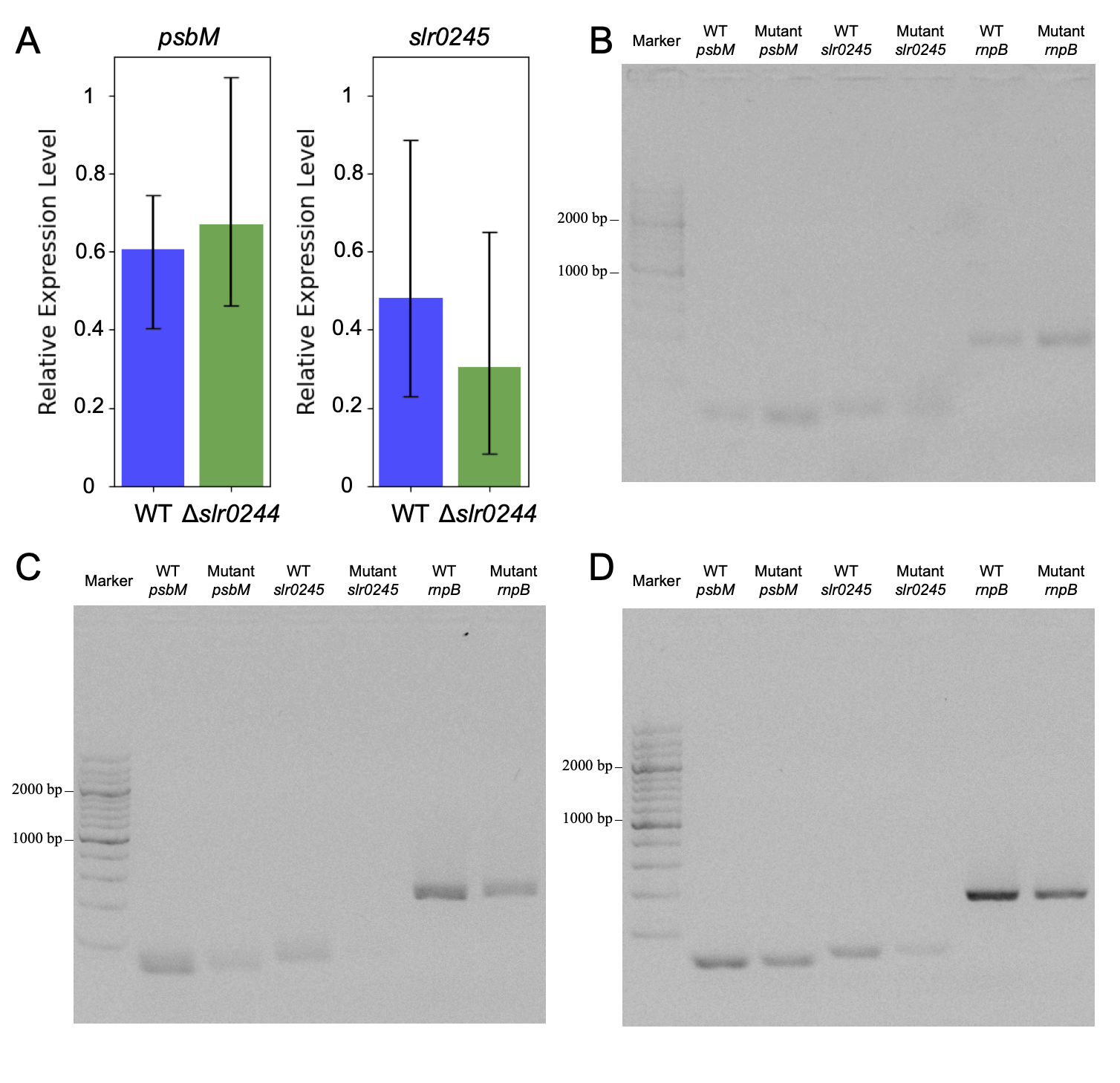
**

**Fig. S4** Expression analysis of *psbM* and *slr0245* in the slr0244 mutant based on semi-quantitative RT-PCR. (A) Average relative expression levels of three experimental results. The error bars indicate the range between minimum and maximum values. (B-D) Gel electrophoresis results for each experiment to calculate the values of panel A.

**
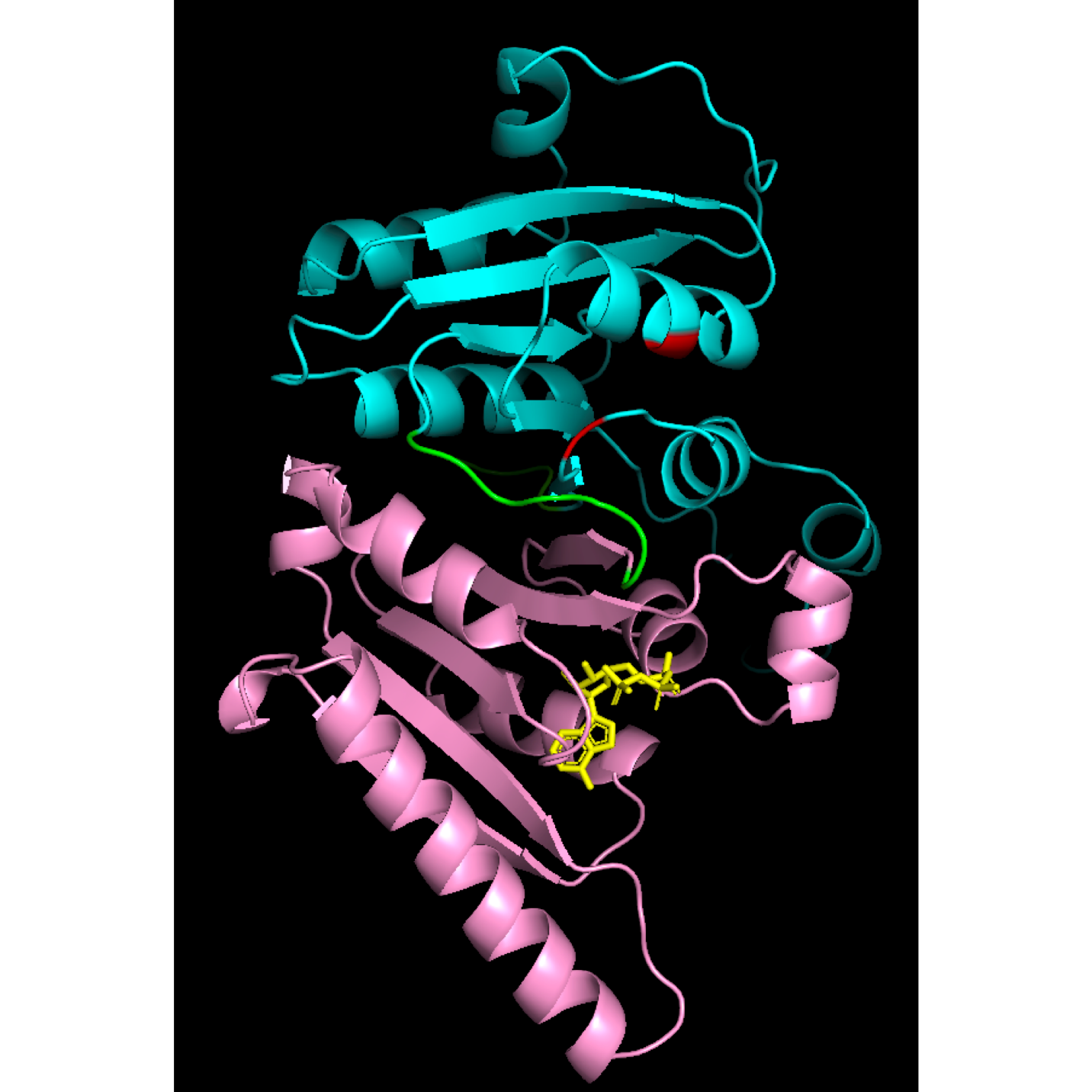
**

**Fig. S5** Prediction of the binding between the Slr0244 protein and ATP by AlphaFold3. The first USP domain is shown in pink, the second USP domain in cyan, other regions in green, ATP in yellow, and the two conserved cysteines in red.

**Table S1** PCR primers for semi-quantitative RT-PCR analysis

| **Target gene** | **PCR product length (bp)** | **Direction** | **Sequence** |
| --- | --- | --- | --- |
| *psbM* | 106 | Forward | 5'- ATGCAAGTTAACAATCTCGGC -3' |
|  |  | Reverse | 5'- AGCTTTCGCTTTGTTTCCC -3' |
| *slr0245* | 140 | Forward | 5'- CGGTGACGTGGAATTTAGAT -3' |
|  |  | Reverse | 5'- GGCTACATCATAGCTCTGGG -3' |
| *rnpB* | 406 | Forward | 5'- GGAGTTGCGGATTCCTGT -3' |
|  |  | Reverse | 5'- TAAGCCGGGTTCTGTTCC -3' |
